# An Ethics Action Plan for Rare Disease Care: Participatory Action Research Approach

**DOI:** 10.2196/46607

**Published:** 2023-11-23

**Authors:** Ariane Quintal, Isabelle Carreau, Annie-Danielle Grenier, Caroline Hébert, Christine Yergeau, Yves Berthiaume, Eric Racine

**Affiliations:** 1 Pragmatic Health Ethics Research Unit Institut de recherches cliniques de Montréal Montréal, QC Canada; 2 Département de médecine sociale et préventive, Université de Montréal Montréal, QC Canada; 3 Ethics and Rare Diseases Working Group, Institut de recherches cliniques de Montréal Montréal, QC Canada; 4 Regroupement québécois des maladies orphelines Sherbrooke, QC Canada; 5 Département de médecine, Université de Montréal Montréal, QC Canada; 6 Division of Experimental Medicine, Department of Medicine, McGill University Montréal, QC Canada; 7 Department of Neurology and Neurosurgery, McGill University Montréal, QC Canada

**Keywords:** community-based participatory research, rare diseases, bioethics, delivery of health care, ethics, clinical, patient participation, empowerment, education, medical, attitude of health personnel, patient education as topic, patient partnership

## Abstract

**Background:**

Owing to their low prevalence, rare diseases are poorly addressed in the scientific literature and clinical practice guidelines. Thus, health care workers are inadequately equipped to provide timely diagnoses, appropriate treatment, and support for these poorly understood conditions. These clinical tribulations are experienced as moral challenges by patients, jeopardizing their life trajectories, dreams, and aspirations.

**Objective:**

This paper presents an ethical action plan for rare disease care and the process underlying its development.

**Methods:**

This action plan was designed through an ethical inquiry conducted by the *Ethics and Rare Diseases Working Group*, which included 3 patient partners, 2 clinician researchers, and 1 representative from Québec’s rare disease association.

**Results:**

The plan is structured into 4 components. Component A presents the key moral challenges encountered by patients, which are the lack of knowledge on rare diseases among health care workers, the problematic attitudes that it sometimes elicits, and the distress and powerlessness experienced by patients. Component B emphasizes a vision for patient partnership in rare disease care characterized by open-mindedness, empathy, respect, and support of patient autonomy from health care workers. Component C outlines 2 courses of action prompted by this vision: raising awareness among health care workers and empowering patients to better navigate their care. Component D compares several interventions that could help integrate these 2 courses of action in rare disease care.

**Conclusions:**

Overall, this action plan represents a toolbox that provides a review of multiple possible interventions for policy makers, hospital managers, practitioners, researchers, and patient associations to critically reflect on key moral challenges experienced by patients with rare diseases and ways to mitigate them. This paper also prompts reflection on the values underlying rare disease care, patient experiences, and health care workers’ beliefs and behaviors. Health care workers and patients were the primary beneficiaries of this action plan.

## Introduction

### Background

A disease is considered rare when its prevalence is estimated to be 1 in 2000 [1]. Owing to their rarity, rare diseases seldom attract the interest of researchers. As a result, their symptoms, mechanisms of action, and therapeutic avenues are poorly covered in scientific literature and clinical practice guidelines [2]. This lack of knowledge often translates into significant difficulties for patients in obtaining timely diagnoses [2]. The prolonged quest for a conclusive diagnosis, known as the diagnostic odyssey, may last several years [3]. Through this process, patients may be misdiagnosed or undergo several inconclusive consultations by specialists [3,4]. This wait is difficult for patients, especially given the lack of satisfactory explanations for their health struggles, and elicits profound uncertainty [5], distress [6], and frustration [7]. Moreover, receiving proper treatment is often impossible for patients with rare diseases; less than 10% of rare diseases benefit from effective treatment [8]. This limited clinical support exacerbates disease manifestations and fuels additional challenges in daily activities, social interactions and relationships, and professional activities [9]. Such difficulties are common to most rare diseases [10] and may lead to moral challenges.

Moral challenges are experiences that are unacceptable in the eyes of patients because they put their values at stake [11] (Racine E, unpublished data, September 2023) and hold great significance, often having profound and multifaceted implications [12]. In health care, diagnostic and prognostic uncertainties, as well as disability, create moral challenges because they impede the ability of patients to pursue important values. These distressing situations put patients’ life trajectories, dreams, and aspirations at stake [13]. Despite the moral distress elicited by these challenges, they have only been superficially investigated and even less so in relation to adults living with rare diseases [14,15]. These salient moral challenges call for ethical inquiries, which involve acquiring an in-depth understanding of moral challenges, critically reflecting on the values they jeopardize, and imagining ways to address them [16,17].

Ethical inquiries are processes by which difficult moral challenges are understood and addressed by discussing possible responses, enacting the most promising response, and evaluating these responses [18]. In other words, ethics and ethical inquiries take moral challenges as objects to ask questions about their nature, value, meaning, and impact in terms of broader notions of fulfillment, such as human development and flourishing. This means that when an experience is designated as being morally problematic (eg, a patient with a rare disease experiencing a situation as a challenge to their own self-esteem or autonomy), this signifies that this experience is lived and experienced as a challenge with respect to one’s values and self-concept, thus calling for a response. Moral experiences are anchored in daily life, including the challenges faced by patients [19]. Moreover, the meaning of these experiences is intrinsically linked to each individual’s unique values and enshrined in the things that matter to them [11,13]. Morality is also social as far as these values are embedded in social practices [20]. Accordingly, the causes of morally problematic experiences can be manifold. It could indeed be the case that there is a questionable attitude from a health care professional causing this problematic experience, but it could also be a simple misunderstanding or miscommunication. This is why ethical inquiries interrogate moral experiences and ask questions about why we experience challenges. Moreover, embedded in this account of ethical inquiry is a distinction between morality as designating the experiential domain of human values and preferences and ethics, which designates a structured field of inquiry, open discussion, research, etc, about tensions and questions raised about human morality and moral experience itself [20,21].

Participatory action research is a promising approach to conducting ethical inquiries that respond to such moral challenges. This approach may be embedded in a diverse range of research methodologies, including qualitative research [22,23]. It differs from conventional research by calling for partnerships with stakeholders from local communities to understand the challenges they face and collaboratively identify ways to mitigate them [22,24]. These challenges may include moral challenges addressed in partnerships with patients. Participatory action research applied to an ethical inquiry is premised on the value of experiential knowledge and agency of patients and other stakeholders [22,25]. Stakeholders may be involved in defining a project’s direction (ie, toward certain moral challenges), improving its methodology, interpreting results, identifying paths for action, and disseminating results [24]. In such projects, ethicists do not position themselves as authoritative experts, dictating how moral challenges should be understood and resolved [16]. Rather than giving their own moral opinions, ethicists provide opportunities for stakeholders to dialogue, engage in these steps of the project, and critically reflect on the values at stake [26]. Therefore, participatory action research in bioethics fosters democratic inquiry, social change, and stakeholder empowerment [22,26].

To the best of our knowledge, few participatory action research projects have been conducted on rare diseases and bioethics. One project, conducted in the United Kingdom, involved the co-design of a national program for genomic screening for rare diseases in newborns with health care professionals, researchers, ethicists, patient groups, and members of the public [27]. Genomic screening for rare diseases raises important ethical issues such as data use and governance, medicalization before illness symptoms, discrimination, and resource use [27]. Otherwise, no participatory project has directly reported an ethical inquiry in relation to the moral challenges experienced by patients and potential solutions that could alleviate these challenges. This gap thus provides an opportunity for methodological innovation in ethical inquiry and an important avenue of investigation for rare disease research.

### Objectives

This paper presents the process and outcomes of the participatory development of an ethics action plan for rare disease care. This action plan represents a toolbox that provides a review of multiple possible interventions for policy makers, hospital managers, practitioners, researchers, and patient associations to critically reflect on the key moral challenges experienced by patients with rare diseases and the ways they could be mitigated. Health care workers and patients are the primary beneficiaries of this action plan, which seeks to promote their ongoing collaboration in health care. This action plan reflects the ethical inquiry introduced above. It provides these actors with an intellectual space to reflect on how rare disease care can be improved, while calling for greater sensitivity to patients’ values. Thus, it departs from traditionally authoritative governmental action plans, whose implementation leads to externally motivated changes in governance, service provision, and resource allocation.

This ethics action plan was developed with members of the rare disease community in Québec, Canada, in accordance with the importance of local contexts for participatory action research [22]. Two bioethics researchers, 3 patient partners, 2 clinical researchers, and a representative from the *Regroupement québécois des maladies orphelines* (RQMO, or Québec Coalition of Orphan Diseases) were involved in the development of the action plan as members of *the Ethics and Rare Diseases Working Group*. The action plan is also supported by scientific literature, gray literature from Québec, and semistructured interviews conducted with patients with rare diseases from this province. This action plan introduces an ethical inquiry into rare diseases that is informative, relevant, and potentially informative in other health care contexts [10]. The participatory process underlying the development of the action plan favors its relevance and usefulness in the lived experiences of patients with rare diseases [22].

This study first describes the participatory process that underlies the development of an action plan. The overall structure of the action plan was then presented. Subsequently, 4 components of the action plan are described: the key moral challenges encountered by patients (component A), a vision for patient partnership in rare disease care (component B), the courses of action prompted by this vision (component C), and promising interventions that could help address the moral challenges by enacting these courses of action (component D). These components, which emerge from qualitative methods, are presented along with commentaries that reflect the perspectives of the working group.

## Methods

### Overview

**Figure 1:**
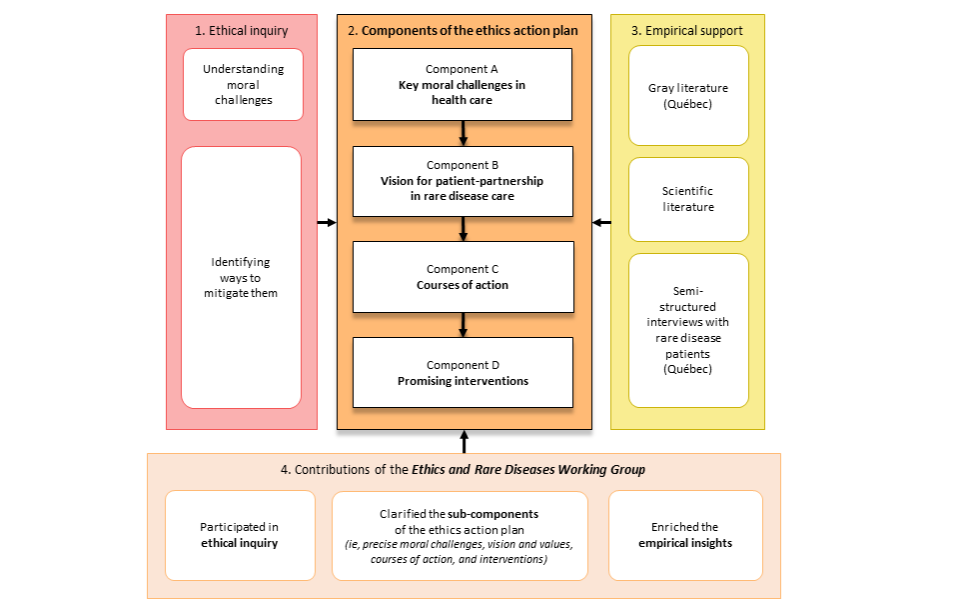
Development of the ethics action plan through ethical inquiry, empirical support, and participatory involvement.

Figure 1 provides an overview of the development of the ethics action plan with the *Ethics and Rare Diseases Working Group* using qualitative methods. The resulting action plan was supported by insights from an exploratory literature review and a community survey conducted by our team [28] (Quintal A, unpublished data, September 2023). The structure of an action plan is inspired by the logic underlying ethical inquiry (Figure 1). It begins by identifying the key moral challenges. It then presents ways to address them through values tied to a vision for patient partnership in rare disease care, courses of action, and promising interventions (Figure 1).

The ethics action plan was developed iteratively by the first author, with significant input from the coauthors. The initial version of the action plan was derived from informally shared insights by working group members during previous meetings held by videoconferences dedicated to parallel qualitative studies [28]. The second and third versions of the action plan were drafted following consultations with the working group held via video conference and email. The action plan was subsequently improved based on the working group’s feedback on manuscript drafts that progressively integrated insights from the exploratory literature review (eg, to further document certain types of interventions) and semistructured interviews (eg, to connect moral challenges and specific components to lived experience; Figure 1). Importantly, and consistent with the distinction drawn above between morality and ethics, a moral challenge is not a label for attribution of blame but rather a concept to designate something that puts at stake one’s deeply held values.

An ethical inquiry approach is central to the development of an action plan. The first author, specializing in ethical deliberation methods inspired by pragmatist ethics [21], intervened as a mediator during consultations with the working group. When disagreements arose during the videoconference consultations, the first author invited each person to expand on their perspective and highlight the importance of the topic. On the basis of the shared information, she proposed a compromise that could be improved through additional discussion. If the disagreement persisted, she prioritized the perspectives of the patient partners, given the research team’s commitment to emphasizing the lived experiences of individuals living with rare diseases. In contrast, no notable disagreements occurred during email consultations as the latter were mostly used to address minor details. Ultimately, the final action plan was approved by all authors via email. Both data collection methods were conducted by the first author with significant input from the coauthors.

An exploratory literature review was conducted to provide empirical support for moral challenges, the vision for patient partnership in rare disease care, courses of action, and promising interventions in the initial versions of the action plan. This exploratory review covered the gray and scientific literature. Consistent with the local scope of participatory research, the gray literature review focused on documents addressing rare disease care, services, and policies in the province of Québec [6,29,30] (personal communication with the RQMO, 2023). Two unpublished documents were included in the gray literature review. The first document, developed in 2013, reports the full results of a provincial survey conducted by the RQMO. The second document, developed in 2017, presents a business plan to guide the expansion of rare disease clinics.

A scientific literature review was conducted through an exploratory approach using PubMed searches mirroring the components of the action plan. Additional scientific literature was consulted to clarify the topics raised in the previously identified literature. To this end, additional PubMed searches were performed, and references cited in the previously identified literature were consulted. All content relevant to the action plan was extracted in a dedicated word document and progressively synthesized in the form of the current article.

Semistructured interviews with adults living with rare diseases [31] were conducted from February to March 2022. The participants were recruited from among the respondents of a previous community survey conducted by our team. Briefly, this survey aimed to document the most morally problematic experiences encountered by individuals living with rare diseases in the province of Québec, Canada. The inclusion criteria for this survey included were being aged 18 years or older, living in Québec when the survey was conducted, and living with a diagnosed or an undiagnosed and suspected rare disease (Quintal A, unpublished data, September 2023). The respondents were recruited through convenience sampling [32]. The RQMO, along with more than 80 patient associations and support groups, was invited to promote the survey in their newsletters and social media pages. Following this effort, 246 questionnaires were initiated, and 121 were sufficiently filled to be included in subsequent analyses. A total of 95 rare diseases were identified among the respondents. Respondents’ ages ranged between 18 and 79 years, and 78.5% (95/121) of the respondents were women (Quintal A, unpublished data, September 2023).

Interview participants were selected from among survey respondents to constitute a diverse sample with regard to age, rare disease, and region. Participants were invited to participate in the interview through an initial short email and were provided with more information, including a consent form, if they expressed curiosity or interest in the study. Among the 18 invited respondents, 12 agreed to participate in the interview. Interviews were conducted by phone or videoconference [33,34]. They were then recorded and subsequently transcribed using an external transcription service.

Simple thematic analyses were conducted on transcript excerpts secondary to the analyses made for another manuscript reporting the primary data (Quintal A, unpublished data, September 2023). Two coding guides were generated. In the first coding guide, which was generated by the first author, primary themes reflected the components of the action plan, which were key moral challenges encountered by rare disease patients (component A), the vision for patient partnership in rare disease care (component B), courses of action (component C), and promising interventions (component D). Secondary themes corresponded to the subcomponents of the action plan [35]. This correspondence was approximately due to the evolving nature of the action plan. Relevant transcript excerpts were linked to secondary themes. The excerpts reported in this manuscript best illustrated these secondary themes. In the second coding guide, which was generated by a research assistant, primary themes and secondary themes were designated categories and subcategories of patient empowerment strategies, respectively. The second coding guide has been validated and enriched by the first author. Component C describes the empowerment strategies uncovered with the second coding guide.

### Ethical Considerations

The study was approved by the Human Subject Ethics Committee of the Montreal Clinical Research Institute (2021-1080). It complied with the Standards on Research Ethics and Scientific Integrity of the *Fonds de recherche du Québec-Santé*, the Tri-Council Policy Statement 2—Ethical Conduct for Research Involving Humans of the Panel on Research Ethics of the Canadian government, and the Helsinki Declaration. The survey respondents provided informed consent before participating in the study. Interview participants were offered $50. Three $50 gift cards were drawn for survey participants. Survey and interview participants were deidentified (ie, by assigning a number to each participant). Transcripts were anonymized (ie, by removing all references to identifiable individuals, locations, etc).

## Results

### Overview of the Ethics Action Plan

**Figure 2:**
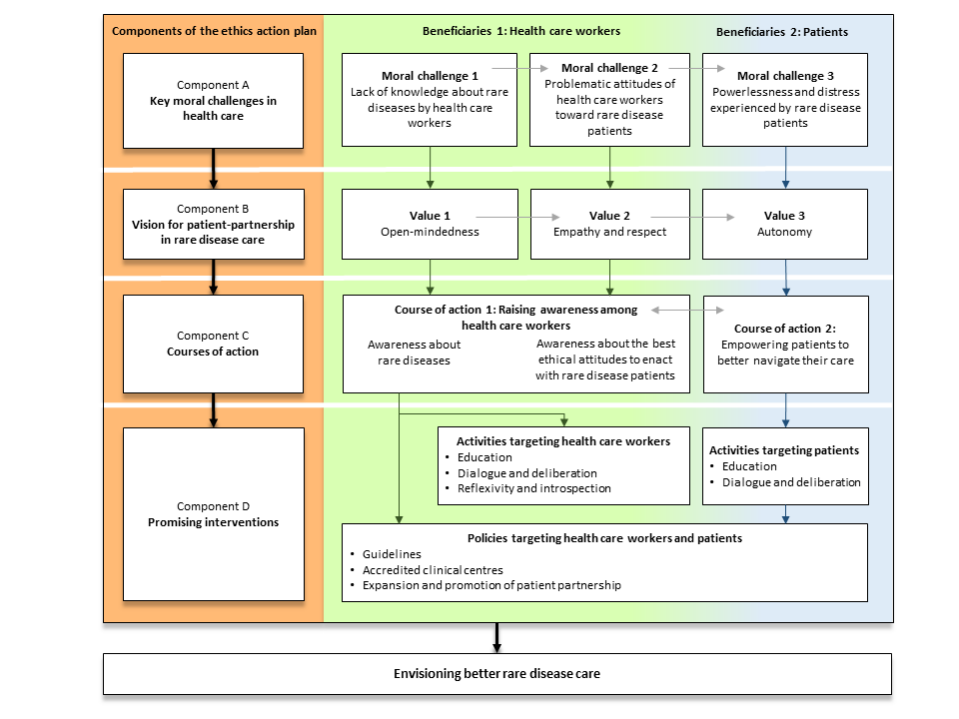
An ethics action plan for rare disease care.

As illustrated in Figure 2, the ethics action plan begins by highlighting the key moral challenges characterizing rare disease care (component A). The lack of knowledge about rare diseases among health care workers may contribute to problematic attitudes toward patients with rare diseases. Following these encounters, patients may feel powerless and distressed. These key moral challenges call for a vision for patient partnerships in rare disease care (component B). This vision is rooted in specific values: open-mindedness from health care workers, empathy and respect from health care workers, and autonomy for patients. Two courses of action target health care workers and patients to move toward these values: greater awareness about rare diseases and patients’ empowerment in navigating their care, respectively (component C). Promising interventions, including activities and policies aimed at health care workers or patients, could foster these dispositions (component D). Ultimately, the ethics action plan aims to stimulate ethical reflection on improving rare disease care through greater sensitivity to patient values and concrete, constructive responses to their needs in health care practice changes.

### Component A: Key Moral Challenges in Health Care

#### Overview

Patients with rare diseases experience a plethora of moral challenges in health care. Three challenges stood out as most morally significant for members of the *Ethics and Rare Diseases Working Group* and for previously collected data [28] (Quintal A, unpublished data, September 2023). These challenges were the lack of knowledge on rare diseases among health care workers, health care workers’ sometimes problematic attitudes toward patients, and the distress and powerlessness experienced by patients (Figure 2; component A). The following paragraphs further describe these challenges with relevant information from existing literature and supporting quotations from the interview participants.

#### Lack of Knowledge on Rare Diseases Among Health Care Workers

Unfamiliarity with rare diseases among health care workers is a key challenge in health care. Health care workers are poorly informed about the manifestations of rare diseases, the available services dedicated to rare diseases, and potential therapeutic avenues when available [29]. This gap in knowledge is unsurprising given that there are between 5000 and 8000 rare diseases worldwide, which have diverse causes and manifestations, including variability within the same rare disease [6,30]. Grasping the breadth and depth of knowledge about rare diseases is an impossible task for health care workers, who have already struggled with limited time and resources. However, health care workers have limited exposure to rare diseases in their training curricula or continuing education [6,29,30]. This could be due to the scattered and scarce scientific expertise on rare diseases [30].

This lack of familiarity with rare diseases among health care workers is a potential cause of the diagnostic odysseys experienced by patients. Misdiagnosis or the absence of a diagnosis may lead to improper medical treatment, unnecessary tests, or exposure to excessive radiation doses due to repeated scans. However, these inappropriate procedures can be expensive, useless, and dangerous [6]. Conversely, a formal diagnosis is usually required to access medications that may halt the progression of a rare disease or alleviate its symptoms. Without proper care, quality of life is compromised, sometimes leading to the death of patients [6,29,30]. Similarly, patients are denied disability benefits without a formal diagnosis [29], increasing financial struggles due to unemployment, and elevated costs of medication, care, and services, for example (Quintal A, unpublished data, September 2023). The multifaceted impacts of diagnostic odysseys elicit psychological distress for patients, adding to the distress they experience due to the lack of explanation for their health condition [6].

#### Health Care Workers’ Sometimes Problematic Attitudes Toward Patients

Previous studies have illustrated that health care workers may display problematic attitudes toward patients with rare diseases. Patients report not being taken seriously [36-39] and express that health care workers do not believe in the severity of their symptoms [10,37,40]. They are sometimes labeled as hypochondriacs [41], or their symptoms are falsely attributed to psychological causes [36,40-42]. Patients are sometimes accused of lying [38,41] or are erroneously told that they invent their symptoms to receive attention or drug prescriptions [38]. Some patients report that health care workers lack compassion [43], belittle and stigmatize them [28] (Quintal A, unpublished data, September 2023), and do not listen to them [36]. Several factors may foster problematic attitudes among health care workers. These attitudes could stem from an unfamiliarity with rare diseases or personal dispositions.

First, health care workers may not be able to recognize their patients’ disease manifestations. Overconfident health care workers may falsely assume that these diseases have psychological causes [44,45]. Alternatively, other health care workers may feel incompetent and resort to inappropriate defense mechanisms. They may project blame, frustration, and anger toward their patients [37,46,47]. Through this resentment, health care workers may also express close-mindedness by refusing to conduct further investigations or learn more about rare diseases [37,38]. By avoiding confidence in rare diseases, health care workers may further reinforce their problematic attitudes.

These factors were intertwined with the problematic attitudes of the participants. Blame, along with disbelief, was central to the testimony of a man aged 32 years living with empty nose syndrome. This condition is a rare and distressing complication of sinus surgery, resulting in loss of sensation of air passing through the sinuses. Upon carefully explaining his situation to numerous physicians, “they all said this: anxiety. They were all blaming it on my mental [state].” Later, he added that physicians told him, “You exaggerate. You are trying too hard. It’s not that severe. You just want to bother everyone with this.”

The 2 interview participants faced close-mindedness. A woman aged 46 years explained, as she sought a diagnosed with Ehlers-Danlos syndrome based on her severe symptoms:

I told [the internist]: “you know, I’m not here to tell you things or teach you stuff, but you likely know that there are many types of Ehlers-Danlos [syndromes].” She told me, ‘Yes, there are six types.’ I look at her, I tell her: “No, there are 14 types. There have been new criteria published in 2017. Would you like to see them?” [She said:] “No, no!” She pushed away my papers.

Later, the participant added that the internist “did not want to learn about my story, my symptoms. She did not want to make a global [assessment of my condition].” A 29-year-old woman living with congenital hyperinsulinism shared a similar story: “among many physicians that I have met in my life, many are close-minded, you know, they are, like, convinced to have absolute knowledge, and they are not really curious to learn more.”

Second, the problematic attitudes sometimes displayed by health care workers may stem from personal dispositions. Some health care workers may be naturally less empathetic than others, and contextual factors may lead them to express these attitudes while facing few negative repercussions. This reality is evidenced in the story of a 47-year-old woman living with Sjögren syndrome secondary to rheumatoid polyarthritis in addition to a suspected disease of the pituitary gland who experienced aggression and violence from a physician [47]. To claim the disability benefits offered by her former employer’s insurance company, she had to undergo evaluation by an expert physician hired as a consultant by the company. The physician was likely to have a conflict of interest: he had a disincentive to recognize her rare disease, as this would be costly for the insurance company that employs him as a consultant. These contextual constraints tainted his attitude toward her, as she explained:

He was aggressive. He manipulated the information. He wouldn’t let me speak. I wasn’t allowed to give information. I couldn’t provide details. I had to answer by yes and no. I was subjected to a list of questions, I could not divert from it. He was aggressive, aggressive towards me. He yelled after me, he raised his voice. He basically kicked me out of his office by saying, “I’m done, you may leave.” Yes, but me, I’m not done. I’m not done.

Such conflicts of interest may arise in other chronic medical conditions or in long-term injuries. However, health care workers with little understanding of rare diseases may erroneously undermine the disability and incapacitation faced by patients and may not know how to properly assess patients with rare diseases. Health care workers risk harming patients if they handle these situations inappropriately, as the woman cited above recalled during her interview.

#### Distress and Powerlessness Experienced by Patients

Medical encounters riddled with tension, such as those described above, leave rare disease patients feeling deeply distressed, worried, and sad [28]. For example, the woman living with Sjögren syndrome described the residual emotional impacts of the consultation with the expert physician: “extremely hurt. Extremely broken. I am extremely disappointed with what I saw and experienced. Very disappointed. Very disappointed with the humanity of these people...It’s a game that is too tough to play for me.”

Through these medical encounters, patients feel profoundly powerless regarding the management of their rare diseases [37,41]. Several factors contribute to this limitation. For instance, patients might not be provided with optimal conditions for shared decision-making [10,38,43]. Often, they are not provided with suitable information regarding rare diseases. According to a survey conducted by the RQMO, 58.8% (144/245) of respondents received little to no information on their rare disease or the rare disease of their relatives (personal communication with the RQMO, 2023). Patients also experience powerlessness when at the mercy of inappropriate care and risk of death due to health care workers’ unfamiliarity with rare diseases [41].

Equally worrisome, patients feel powerless regarding their illness management due to the internalized skepticism expressed by health care workers. Following the problematic attitudes of health care workers, patients may begin to doubt their illness experiences, symptoms, and rationality [37,40,48]. A woman living with Sjögren syndrome explained: “When you are faced with cruelty, you are screwed. Being yelled at, being ridiculed, and being laughed at in my face, it’s like...it destroys you so much! This destroys you very much. You question yourself.” This powerlessness may be enhanced by an uncertain prognosis, which often accompanies rare diseases [41]. Through the accumulation of these experiences, patients may ultimately lose trust in the ability of the medical establishment to provide appropriate care [10,37,41,49].

### Component B: Vision for Patient Partnership for Rare Disease Care

#### Overview

In response to the challenges presented above, members of the *Ethics and Rare Diseases Working Group* proposed the vision of patient partnership in rare disease care. Patient partnerships bridge the medical and scientific knowledge of health care workers with the experiential knowledge of patients [25,50]. Patients acquire experiential knowledge through their first-hand experiences of their medical conditions, care trajectories, disability, occupational hardships, and associated psychosocial repercussions [25,51]. Many patients also acquire theoretical knowledge by consulting the medical literature on rare diseases or by attending conferences [52]. This study compensates for the limited information they receive from health care workers (personal communication with the RQMO, 2023).

Through patient partnerships in care, health care workers value patients’ competencies and judgments [53]. Patient partnership in care contrasts with older models of care, where patients are merely provided with medical information and are involved by health care workers in care decisions [54,55]. In this approach, health care workers genuinely consider patients’ perspectives and experiences rather than merely listening to them [52]. They support patients in making health-related decisions, managing their health autonomously, and acquiring health-related responsibilities in ways that are compatible with their needs and personal inclinations [50,52,53,56,57].

Working group members envisioned 3 guiding values for patient partnerships to respond to the 3 moral challenges previously identified. These values were open-mindedness from health care workers, empathy and respect from health care workers, and autonomy of patients (Figure 2; component B). These values could also work synergistically; positive attitudes of health care workers are likely to be conducive to the expression of patient autonomy. A 61-year-old woman living with chronic myelogenous leukemia explained: “communication and teamwork, partnership; in fact, this was a success. It will help the patient recover more quickly, leave the hospital more quickly, and have a better quality of life.” In the next sections, these 3 values are described with supporting information from the literature and semistructured interviews.

#### Open-Mindedness of Health Care Workers

As a value, open-mindedness could help health care workers overcome their lack of knowledge of rare diseases. Patient partnership may only be achieved with this open-mindedness of the patients’ experiential knowledge [49]. Through greater open-mindedness, health care workers may more readily suspect rare diseases, educate themselves on rare diseases, and believe in patients’ experiences.

With greater open-mindedness, health care workers would adopt a “pedagogy of doubt,” a disposition promoted by French rare disease associations [30,58]. Health care workers can suspect rare diseases when faced with patients who exhibit atypical disease manifestations with no apparent cause. They could conduct further investigations into rare disease diagnoses rather than quickly dismissing patient complaints. They could refer these patients to specialists who can readily diagnose them under appropriate conditions [30]. The 32-year-old man cited above living with empty nose syndrome explained: “take the time to reject all [disease] possibilities. If you have not rejected them and you send [the patient] to the psychiatrist, the physical [ailment] is still here, but [they] are not in the right place. [They] are not at the right specialist.” In addition to allowing for proper care, such investigations are important given the epistemic value of diagnoses for patients [59]. Diagnoses validate patients’ experiences [60] while helping them make sense of their confusing and distressing symptoms [37].

With greater open-mindedness, health care workers are also more receptive to listening to patients. From patients’ testimonies, health care workers can learn about rare diseases and their psychosocial impact. A 40-year-old woman living with hereditary angioedema echoing the suggestions of 2 other participants explained the following:

Listening is the most important factor...Rare diseases...are so rare that it is possible that you never encounter them in your life, so it is normal that [healthcare workers] do not know them. However, listen to the patient, at least! First, the patient who specifically has a rare disease, their disease, they will know it from A to Z. [The patient] will be able to explain the entire inflammatory process if needed...I am able to explain entirely what happens in my body. Listen!

Open-minded health care workers are also more likely to believe patients’ narratives. This was the main recommendation put forward by a 47-year-old woman living with Nutcracker syndrome in addition to other rare diseases: “I would simply like them to start by believing us.”

This does not necessarily imply that health care workers should uncritically believe patients. Rather, health care workers should be attentive to the hardships expressed by the patient with a humane and humble demeanor. To their best ability, health care workers should value patients’ perspectives and experiential knowledge. Health care workers should avoid disbelieving, stigmatizing, or belittling patients. Exchanges between health care workers and patients should be constructive and bidirectional, meaning that patients should approach the former with a positive attitude. Patient knowledge, which may align with medical knowledge, may help health care workers compensate for their lack of knowledge.

Often, health care workers’ tendency to identify a given condition is influenced culturally. For example, despite its overwhelming rarity [61], psychosomatization is often erroneously suspected in patients [30]. This cultural bias is reflected by a recent issue dedicated to “disorders of somatic symptomatology” in *Le Médecin du Québec* in 2021, a medical magazine published by the *Federation of General Practitioners of Québec* [62]. Open-mindedness could guide health care workers to unpack biases that inform their practice. A 55-year-old woman with mast cell activation disorder, a lupus-like syndrome, and severe postviral syndrome resulting from COVID-19 emphasized the need for a culture change among health care workers. She critiqued their appeal to unfounded judgments and beliefs when evaluating patients rather than a rational and evidence-based approach.

#### Empathy and Respect of Health Care Workers

The value of greater empathy and respect could be fundamental for rare disease care [39]. Empathic health care workers are more readily able to imagine themselves in the situations of their patients and are concerned about their well-being [63-65]. During respectful encounters, health care workers value patients, give them adequate attention, and demonstrate esteem, courtesy, and consideration [66-68]. Without empathy and respect from health care workers, patients may not feel supported in making health decisions [49]. The importance of empathy and respect has been highlighted in the Québec government’s recent Rare Disease Policy [6]. Empathy and respect can manifest as compassion, solidarity, kindness, or courage depending on the context.

For example, in our interviews, several participants expressed that they would appreciate more compassion from health care workers. A 31-year-old woman living with granulomatosis and polyangiitis explained that “we feel so vulnerable, I think that I would only need a little bit of human warmth, for real.” She added that if health care workers are unable to express compassion at the patient’s bedside, they could be accompanied by a nurse or social worker when announcing a life-changing diagnosis. Moreover, the 55-year-old woman cited previously emphasized the importance of cultivating courage among health care workers, which is essential to kindness and respect:

[We] have to explain to medical students that it’s their duty to be here, even when it is difficult...It’s part of their job to accompany human suffering. You can’t want the patient to be somebody else’s problem because it’s a difficult case...So if [the patient] has the courage to be there, the healthcare professional should have the courage to be there as well.

#### Autonomy of Patients

In alignment with this value, patients with rare disease can be supported in making health decisions autonomously [50]. Decisions made autonomously by patients reflect their personal preferences, converge toward their goals and aspirations, are contingent on the information at hand, and are grounded in their life trajectories [69]. Helping patients achieve a high level of autonomy in disease management is a key objective of patient partnerships in care [50].

Valuing autonomy does not equate to holding patients fully responsible or worse, blaming them [70,71]. Rather, health care workers could recognize that autonomy is contextual, meaning that certain conditions may promote or inhibit a patient’s capacity to act autonomously [69]. Health care workers may promote patient autonomy by creating a care setting conducive to these abilities [71,72]. For example, some conditions may be conducive to decisions that are voluntary (ie, free of coercion), deliberate (ie, supported by arguments open to critical examination), and supported by sound information [69]. A previously cited 29-year-old woman living with congenital hyperinsulinism lamented that the physicians who cared for her during her pregnancy were not familiar with her rare disease and gave her contradictory treatment advice. As a result, she could not fully enact her autonomy. She explained it as follows:

Well, first, I felt not being able to make an informed decision regarding [2 competing medications], since I had the impression that I was missing information and that the physicians provided contradictory information...As a result, you know, I did not have the opportunity to have a preference...for my decision.

### Component C: Courses of Action

#### Overview

The *Ethics and Rare Diseases Working Group* reflected on courses of action that would cultivate approaches that reflect the vision and values discussed previously in response to the key moral challenges identified. The first course of action would be to raise awareness among health care workers regarding rare diseases and appropriate ethical attitudes to adopt in rare disease care. A second course of action would be to empower patients to navigate their care better.

#### Raising Awareness Among Health Care Workers

In accordance with the first course of action, health care workers could be sensitized to the existence of rare diseases and appropriate ethical attitudes to adopt in rare disease care. This strategy could foster a context favorable to patient partnership in rare disease care by proactively defusing tensions inherent to clinical encounters in the context of rare diseases.

First, this course of action could address issues of misdiagnosis and disbelief by exposing health care workers to the idea that rare diseases truly exist and may be present among their patients. Awareness efforts could emphasize the diversity of rare diseases, their varied clinical presentations, and their often multisystemic repercussions. They could also showcase the usual life-altering consequences of living with a rare disease, ranging from psychosocial challenges to financial hardships [6]. Raising awareness could also reveal the ways in which these struggles are exacerbated by diagnostic odysseys and a lack of recognition from health care workers.

Efforts to raise awareness could also include providing health care workers with high-quality sources of information on rare diseases [6]. These sources include materials curated by scientific consortia (eg, the Orphanet portal) [6], research institutes (eg, the National Institutes of Health’s Genetic and Rare Diseases Information Center) [73], and rare disease associations (eg, RQMO’s iRARE Centre) [74]. By consulting these resources when faced with atypical patients, health care workers can progressively learn more about rare diseases.

These strategies to raise awareness could likely cultivate greater open-mindedness among health care workers and counter their lack of knowledge. Perhaps this could prove more effective than familiarizing health care workers with various training opportunities for rare disease characteristics they may quickly forget.

Second, health care workers can become acquainted with the life-changing impacts of their attitudes on patients. Beyond empathy and respect, these attitudes could also encompass compassion, solidarity, kindness, courage, and greater sensitivity to the peculiarities of rare disease patients’ situations [6]. Health care workers could also be made aware of the distress and powerlessness experienced by patients, which often results from encounters where empathy and respect are lacking. When conveying these attitudes to health care workers, special attention should be paid to their expertise to avoid patronizing them.

Raising awareness about rare diseases and important approaches to rare disease care among health care workers also coincides with Axis 1 of the Québec government’s Rare Disease Policy. This axis emphasizes the need to create or improve awareness of rare diseases among health care workers through training, knowledge translation, and improved access to information [6].

#### Empowering Patients to Better Navigate Their Care

The second course of action consists of empowering patients to better navigate the health care system and manage their health. Empowerment is the ability to take responsibility and exert control in decisions and actions regarding one’s life, in the spirit of self-determination [71,75-78]. In health care settings, empowered patients seek information on rare diseases and health care systems. They make medical decisions by mobilizing appropriate resources and subsequently reflecting on the outcomes of these decisions [76,77].

Through their struggle for recognition, individuals living with rare diseases are known for their empowerment and resilience [79]. Paradoxically, quotations from the interview participants suggest that feelings of powerlessness coexist with empowerment strategies. It is plausible that the latter varies across individuals based on their personal dispositions and rare diseases. Thus, patients can plausibly benefit from the diverse empowerment strategies used by others. Consistent with the tenets of patient partnership in care, empowering patients can help them overcome their feelings of distress and powerlessness while reinforcing their autonomy [29,80]. Patient empowerment is also fostered by health care workers recognizing patients’ expertise [81,82], supporting the expression of their preferences and autonomy [72,82], and providing them with the necessary resources to make health choices [71].

Following this course of action, patients could be exposed to empowerment strategies such as those uncovered in the survey study and interviews conducted by our research team. These strategies, which pertain to health care settings or personal health management, are summarized in Table 1 [28]. However, this does not constitute a comprehensive portrayal of all the empowerment strategies that may be used by patients. Patient empowerment aligns with Axis 3 of Québec’s Rare Disease Policy. Axis 3 highlights the importance of promoting research and innovation in the realm of rare diseases, notably through knowledge translation initiatives between researchers, health care workers, and patients, as exemplified in Table 1 [6].

**Table 1:** Patient empowerment strategies for health care settings.

Categories and subcategories of strategies^a^			Strategies evidenced in a previous survey study and interviews on rare diseases [28]
**Patient capacities, states, and resources**			
	Improving health literacy	Seeking information on rare diseases through web searches or with the help of rare disease associations	
	Skills and attitudes symbolic of autonomy in care	Vigilance regarding treatments administered, especially during hospitalizations Being resilient Being combative about one’s health Being perseverant	
	Control over health and care	Returning to the hospital to seek answers Being accompanied by a close one or a representative of the hospital’s users’ committee	
**Patient behaviors**			
	Seeking medical care	Turning to private health care Contacting international experts or traveling to obtain treatment, sometimes with the help of a GoFundMe campaign Consulting several health care workers despite long waiting times Privileging consultations with open-minded health care workers Being selective about the hospital to travel to Seeking a second medical opinion	
	Taking an active role in health care consultations	Bringing written personal information to health care workers (eg, medical history, symptoms, possible diagnoses, and recommended and contraindicated drugs), while avoiding sharing information that could fuel their prejudices Bringing scientific documentation to health care workers Writing a letter to a specialist Discussing medical interventions with health care workers Looking for other medical treatments	
	Health self-management	Carefully managing one’s rare disease and symptoms Using various strategies to access medical devices Developing a protocol for one’s rare disease Complying with a prescribed treatment despite doubts about its appropriateness	
	Making medical decisions	Choosing to opt out from a prescribed treatment Opting out of medical consultations due to dissatisfaction	
	Seeking reparation	Lodging complaints	

^a^Both are inspired by the framework on patient empowerment developed by Bravo et al [77] and the literature cited in this paper.

### Component D: Promising Interventions

#### Overview

The previously proposed courses of action, which raise awareness among health care workers and foster patient empowerment, may be realized through various interventions. Interventions are classified as activities targeting health care workers or patients or as policies based on a generous interpretation of a typology coined by Michie et al [83,84]. A critical analysis of the most promising interventions based on ideas from the *Ethics and Rare Diseases Working Group* and insights from the literature is presented below. This action plan represents a toolbox that provides a review of multiple possible interventions for policy makers, hospital managers, practitioners, researchers, and patient associations to develop context-appropriate interventions for improving rare disease care.

#### Promising Activities for Raising Awareness Among Health Care Workers

Health care workers could be sensitized to the existence of rare diseases and educated on appropriate ethical attitudes to enact with patients through activities related to education, dialogue, deliberation, or reflective activities (Table 2). These activities can be organized by ethicists, social workers, social scientists, health care workers, and, sometimes, in partnership with patients.

**Table 2:** Promising activities to raise awareness among health care workers.

Type of activity and promising activities		Description	Limitations	Benefits
**Educational activities (examples focus on continued education)**				
	Training activity with an evaluative component	Professors, patient representatives, or patients would assess the ethical attitudes of health care workers.	Knowledge and practices of primary care physicians may not improve following patient feedback [84-88].	Enrollment is incentivized by the scarcity of continuing education credits with an assessment component [89].
	Talk by patients	Patients would share challenging or positive experiences while emphasizing the need for appropriate ethical attitudes.	Patients may feel uncomfortable sharing negative experiences.	Such talks may capitalize on existing activity with regular attendance such as clinical grand rounds [90].
	Talk by a health care worker living with a rare disease	A health care worker living with a rare disease would sensitize health care workers to rare diseases and the need for appropriate ethical attitudes.	The health care worker may face professional prejudice by exposing a personal vulnerability.	Knowledge uptake may be more effective through a talk led by a health care worker as opposed to a patient. The former may bypass the prejudice of his or her peers.
	Article followed by training session	A short article published in the newsletter of a professional medical association could raise awareness on rare diseases and advertise an upcoming webinar on appropriate ethical attitudes.	Health care workers may only skim through the newsletter, limiting its ability to raise awareness.	Professional medical associations have wide readership, favoring good exposure to such articles.
**Dialogue and deliberation**				
	Collaborative learning	A group of health care workers, which possibly includes patients, would meet regularly [91]. At each meeting, an alternating health care worker would describe complex situation involving a rare disease patient. Others are invited to provide input on the case [92].	These activities require regular time commitments and are difficult to scale. These activities may not interest the health care workers which exhibit the most problematic attitudes.	Collaborative learning fosters critical reflection, mutual learning, and improved understanding, especially with patient involvement [91,93].
	Pairing programs	Health care workers would be put in relation with rare disease patients and their families. They would exchange freely with them outside of health care settings during a few meetings [94].	Same as previous	Health care workers may become more confident and less anxious to interact with rare disease patients [63,95,96].
**Reflective activities**				
	Moral case deliberation	A team of health care workers would reflect on the moral disagreement or uncertainty they face together in a situation [97,98] involving a rare disease patient. An ethicist would help them to recognize the values at play and identify solutions [97-99].	The ethicist only initiates this process if prompted by health care workers. Yet, the latter may not be fully sensitive to moral disagreement or uncertainty.	This activity fosters moral competency [97,98].
	Debiasing activities	These activities aim to reduce biases [63,100,101]. Health care workers would reflect on how they would act in fictive situations involving rare disease patients. Then, they would be exposed to appropriate ethical attitudes [63], prompting them to critically reflect on their biases and attitudes [63,102].	Health care workers which hold the most biases are the least likely to enroll in the activity.	Debiasing may be very effective in fostering changes by eliciting strong emotional reactions (eg, shame) [62,101].
	Autoethnography	A few health care workers would write personal narratives about difficult situations they faced with rare disease patients. They critically analyze their narratives through qualitative methods [103,104].	Autoethnography is particularly time- and resource-intensive. Insights may not be entirely transposable to future situations.	If presented during a clinical grand rounds session [104], the narratives benefit the audience in addition to those who have undertaken the autoethnography.

First, educational activities may be delivered through a variety of formats, namely, webinars, workshops, written materials, and sessions within clinical grand round schedules [90]. These activities could be offered at various training stages, such as during entry-level studies [29,30], residency [30] (personal communication with Yves Berthiaume, 2023), or as continuing education [30]. Activities offered as continuing education may more readily counter systematic misunderstandings or prejudice disseminated over time through the hidden medical curriculum [104-106]. In particular, continuing education has been shown to improve knowledge among general practitioners [84,107]. Four promising continuing education activities are listed in Table 2.

Second, activities centered on dialogue and deliberation may provide spaces for health care workers to learn together. Alternatively, they may provide an opportunity to learn from patients without constraints in the clinical setting. Unlike educational activities, they require periodic involvement instead of being offered as a single session. Two promising dialogic and deliberative activities include collaborative learning and pairing programs (Table 2).

Third, during reflective activities, health care workers would “explore or examine a situation, an issue, or a particular object on the basis of their past experiences to develop new understandings that will ultimately influence their actions [and] challenge the practices, roles, beliefs, and values of practitioners” [108]. Difficult situations experienced by patients with rare diseases can be targeted through these reflective activities. Reflection is central to 3 promising activities: moral case deliberation, debiasing, and autoethnographies (Table 2).

#### Promising Activities for Fostering Patient Empowerment

Patients learned empowerment strategies through activities related to education, dialogue, and deliberation (Table 3). These activities could feature the empowerment strategies discussed in Table 1, or rare disease patients could share their personal strategies. In Québec, patient empowerment activities would benefit from being delivered in French to compensate for the lack of rare disease information offered on the internet in this language [30]. These activities could be designed or facilitated by patient experts, patient association representatives, ethicists, or social workers.

**Table 3:** Promising activities to foster patient empowerment.

Type of activity and promising activities			Description		Limitations		Benefits
**Educational activities**							
	Short explanatory documents	These documents would introduce empowerment strategies with illustrations, diagrams, and positive language. They could be shared on social medial, in newsletters, or through pamphlets distributed in waiting rooms.		Efforts needed to diffuse these documents should not be underestimated.		They can reach many patients.	
	Short informational videos	These videos would use clear visuals and concise language to discuss empowerment strategies. They could be shared on social media and newsletters.		More resources are required to develop videos than documents.		Same as previous.	
	Detailed articles	Detailed articles addressing empowerment strategies would be published in patient-oriented magazines. An example is the *Magazine Expériences* in Québec [109], a magazine produced by patient partners.		These specialized magazines have a limited readership.		Such articles enable patients to share their empowerment strategies and conversely, allow readers to become familiar with them.	
	Workshops	Workshops, offered virtually or in-person, would expose patients to documented empowerment strategies. They could include a subsequent breakout session to allow patients to share their personal strategies.		No limitations identified.		Workshops integrating theoretical content and patient interactions are informative and dynamic; web-based workshops accommodate geographically dispersed patients or those living with physical limitations.	
**Dialogue and deliberation**							
	Regular empowerment meetings	Patients would discuss empowerment strategies during regular group meetings. They would update others on their use of these strategies in ongoing situations.		Such meetings require regular time and energy investment from patients.		This activity provides a safe space for discussion among patients. It builds capacity and cooperation.	
	Patient companionship	Undiagnosed rare disease patients are paired with diagnosed patients. Pairs would be prompted regularly to discuss alternating empowerment strategies.		Discussions may drift away from empowerment as they occur privately within the pairs; this mentorship dynamic may be demanding for the experienced patient.		This activity fosters close relationships centered on peer support.	

First, educational activities can be implemented or advertised by rare disease associations, moderators of online support groups, or health care workers. These activities include short explanatory documents, short informational videos, detailed articles, and workshops (Table 3). Second, activities centered on dialogue and deliberation could be developed through partnerships between various professionals, such as social workers, occupational therapists, or ethicists, and patient associations or moderators of online support groups. Dialogue and deliberation are explicitly directed toward the topic of empowerment, thereby surpassing the scope of existing group and peer support initiatives. These activities could preferably be offered virtually to accommodate the geographic dispersion or physical limitations of the patients. Regular empowerment meetings and patient companionship activities are examples of these activities (Table 3).

The patient empowerment activities described in Table 3 overwhelmingly emerged from the discussions with the *Ethics and Rare Diseases Working Group*. Most patient empowerment activities described in the literature aim to improve illness management and clinical outcomes [82,110,111] (eg, therapeutic educational programs in France [30]) rather than to empower patients more holistically in their daily lives.

#### Promising Policies and Public Actions for Raising Awareness Among Health Care Workers and Fostering Patient Empowerment

Novel policies and public actions should be implemented to raise awareness among health care workers and promote patient empowerment. Policy development requires more resources than activities that target health care workers and patients. Nonetheless, promising policies include the development of guidelines for rare disease care, creation of accredited clinics, and expansion and promotion of patient partnerships.

First, a guideline document addressing the commonalities between most rare diseases could present the common challenges encountered by patients, emphasize the often unusual clinical presentations of rare diseases, and introduce appropriate ethical attitudes to enact with these patients [6]. These guidelines could also direct health care workers toward high-quality sources of information on rare diseases (Figure 2; course of action 1). These guidelines could also include references to local patient associations and online support groups [39]. With these guidelines, health care workers would be better equipped to direct their patients toward appropriate resources, as they undergo more detailed clinical investigations.

Second, accredited clinical centers for rare diseases could be created to unite several clinicians, benefiting patient care and interprofessional collaboration. Clinical centers could receive accreditation provided that their health care workers receive proper training on rare diseases and on appropriate ethical attitudes, notably through the above-mentioned educational activities. Despite being resource-intensive, this accreditation strategy would ensure the uptake of appropriate ethical attitudes among health care workers employed in these clinics and favor high-quality care [30].

Third, patient partnerships should be expanded and promoted for policy making and research. Such approaches contrast slightly with the vision of patient partnerships in care. Patient partnership in policy making or research involves integrating patients into management teams or research groups [30,51,53] beyond health care settings. Patient partners provide valuable inputs on paths for improving health care and health services. They also contribute to drafting policies and guidelines by mobilizing experiential and theoretical knowledge [30,51,112]. Although patient partnerships in research and policy making are gaining traction in Québec [112,113], they warrant greater extension to rare disease patients. As patients are not always knowledgeable about patient partnership initiatives, simple interventions could be implemented to enhance their exposure to these patient partnership opportunities, notably through flyers made available in waiting rooms or web-based advertisements. Although patient partnerships may require additional resources, the expansion and promotion of patient partnerships could help counter the marginalization experienced by patients with rare diseases. Our open experience in doing this is telling in terms of the ability of such partnerships to carry our research much further.

## Discussion

This paper presents a first ethical action plan for improving the quality of care offered to individuals living with rare diseases. The action plan is articulated around 4 components: the key moral challenges encountered by patients (component A), a vision for patient partnership in rare disease care (component B), courses of action prompted by this vision (component C), and promising interventions that could help address moral challenges by enacting these courses of action (component D). It is intended to serve as a resource and toolbox to help policy makers, hospital managers, practitioners, researchers, and patient associations critically reflect on the key moral challenges experienced by this patient population and on ways to address these challenges. This action plan was supported by insights from the *Ethics and Rare Diseases Working Group*, gray and scientific literature, and semistructured interviews. More broadly, the article reflects on the values underlying rare disease care and patient experience, in addition to health care workers’ beliefs and behaviors.

Although this ethical action plan is anchored in sociocultural elements specific to Québec, the ethical inquiry it initiates may benefit other health care contexts and cultures. Hence, future research conducted in Québec or elsewhere could involve the design, implementation, and evaluation of the promising interventions identified in this study. These activities can be developed in collaboration with working groups comprising stakeholders and should be subjected to responsive evaluations. These evaluative processes, which are participatory in nature, aim to assess the value of clinical practices or programs and to identify potential improvements [114,115]. The political landscape in Québec, which is characterized by the recent publication of a governmental policy for rare diseases [6], will likely favor the dissemination of this ethics action plan and the development of such activities.

## Abbreviations

RQMO: Regroupement québécois des maladies orphelines (Québec Coalition of Orphan Diseases)
